# Supplementary material for: CD34+ Cell Dose, Measurable Residual Disease, and Outcome After Myeloablative HLA-Matched Peripheral Blood Hematopoietic Cell Transplantation for Adults with Acute Myeloid Leukemia
Source: Cancers (Basel). 2025 Jul 12;17(14):2323. doi: 10.3390/cancers17142323 (PMC12293095; doi:10.3390/cancers17142323)
Supplement: Supplementary file 1 [file cancers-17-02323-s001.zip › cancers-3714842-supplementary.pdf]

Supplemental Table S1. Patient characteristics stratified by high/low CD34+ cellular dose groups.

	CD34+ high (>5.61x10^6/kg), n=277	CD34+ low (≤ 5.61x10^6/kg), n=81	*P*-value
Age, median (range)	50 (18-74)	51 (18-69)	0.49
Body weight, in kg (range)	78.0 (47.3-187.5)	83.7 (48.2-176.0)	**0.025**
Male sex, n (%)	142 (51.3%)	42 (51.9%)	0.93
HCT CI, n (%)			0.84
Low (0-1)	128 (46.2%)	35 (43.2%)	
Intermediate (2-3)	91 (32.9%)	26 (32.1%)	
High (≥4)	53 (19.1%)	19 (23.5%)	
Unknown	5 (1.8%)	1 (1.2%)	
ECOG PS, n (%)			**0.0020**
0	103 (37.2%)	15 (18.5%)	
1-2	174 (62.8%)	65 (80.2%)	
Unknown	0 (0%)	1 (1.2%)	
TRM, median (range)	1.1 (0.04-17.4)	1.6 (0.1-6.2)	**0.0093**
Cytogenetic risk, n (%)			0.61
Favorable	10 (3.6%)	1 (1.2%)	
Intermediate	172 (62.1%)	55 (67.9%)	
Adverse	85 (30.7%)	23 (28.4%)	
Unknown	10 (3.6%)	2 (2.5%)	
Secondary AML	58 (20.9%)	14 (17.3%)	0.47
Blood count before HCT, n (%)			0.16
CR	209 (75.5%)	64 (79.0%)	
CRh/CRi	56 (20.2%)	17 (21.0%)	
MLFS	12 (4.3%)	0 (0%)	
Karyotype before HCT, n (%)			0.23
Normalized	111 (40.1%)	24 (29.6%)	
Abnormal	52 (18.8%)	17 (21.0%)	
Non-informative*	114 (41.2%)	40 (49.4%)	
Year of HCT, n (%)			0.56
2006-2017	182 (65.7%)	56 (69.1%)	
2017- 2023	95 (34.3%)	25 (30.9%)	
Donor age, median (range)	35 (17-70)	37 (19-69)	0.23
Donor type, n (%)			0.84
Matched sibling donor	106 (38.3%)	30 (37.0%)	
Matched unrelated donor	171 (61.7%)	51 (63.0%)	
Graft status			0.42
Fresh	236 (85.2%)	66 (81.5%)	
Cryopreserved	41 (14.8%)	15 (18.5%)	
GVHD prophylaxis, n (%)			0.68
CNI+MMF±Sirolimus	50 (18.1%)	10 (12.3%)	
CNI+MTX±Other(s)	199 (71.8%)	62 (76.5%)	
PTCy	24 (8.7%)	8 (9.9%)	
Others	4 (1.4%)	1 (1.2%)	
Maintenance therapy, n (%)			0.29
None	202 (73.0%)	54 (66.7%)	
FLT3 inhibitor	17 (6.1%)	4 (4.9%)	
HMA +/- VEN	5 (1.8%)	0 (0%)	

HMA + FLT3 inhibitor	1 (0.4%)	1 (1.2%)	
Unknown	52 (18.7%)	22 (27.2%)	

*Normal cytogenetics in patient with cytogenetically normal AML or missing cytogenetics at diagnosis. Abbreviations: CNI, calcineurin inhibitor; CR, complete remission; CRh, CR with partial hematologic recovery; CRi, CR with incomplete hematologic recovery; GVHD, graft-versus-host disease; HCT, hematopoietic cell transplantation; HLA, human leukocyte antigen; MLFS, morphologic leukemia-free state; MMF, mycophenolate mofetil; MRDpos, measurable residual disease positive; MRDneg, measurable residual disease negative; MTX, methotrexate; PTCy, post-transplantation cyclophosphamide; TRM, treatment-related mortality

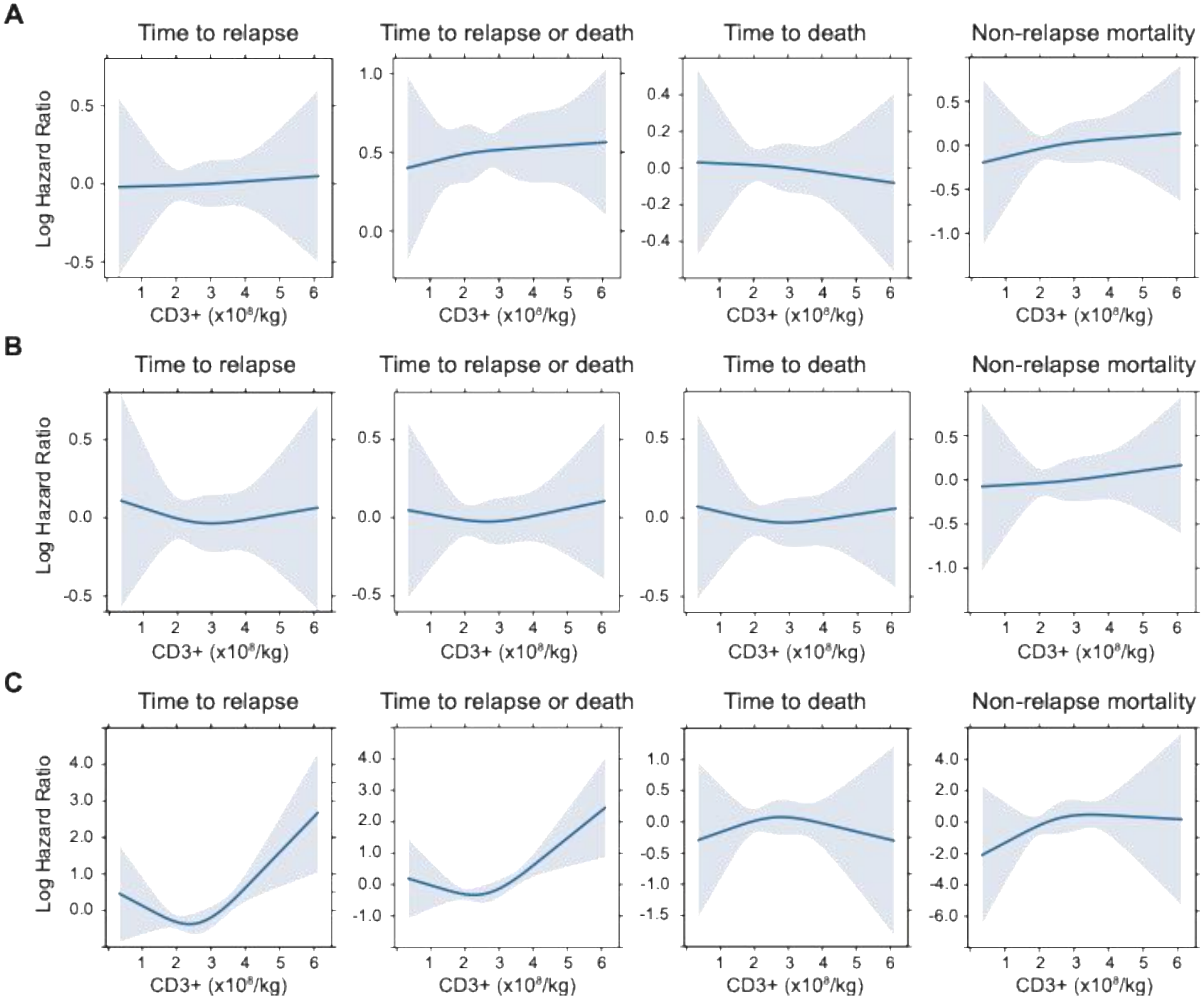

Supplemental Figure S1. Restricted cubic spline functions for post-HCT outcomes of relapse, relapse-free-survival, overall survival, and non-relapse mortality in (A) the entire cohort, (B) the MRD^{neg} cohort, and (C) the MRD^{pos} cohort based on CD3+ dose.
